# Design, development and preclinical assessment of MENAVip-ICP, a new snake antivenom with potential coverage of species in the Middle East and North Africa regions

**DOI:** 10.1016/j.toxcx.2024.100206

**Published:** 2024-08-30

**Authors:** Álvaro Segura, Edwin Moscoso, Deibid Umaña, Mariángela Vargas, Andrés Sánchez, Andrés Hernández, Gina Durán, Mauren Villalta, Aarón Gómez, María Herrera, Mauricio Arguedas, José María Gutiérrez, Guillermo León

**Affiliations:** Instituto Clodomiro Picado, Facultad de Microbiología, Universidad de Costa Rica, San José, Costa Rica

**Keywords:** MENAVip-ICP, Snake venom, Snake antivenom, Preclinical efficacy, Snakebite envenoming, North Africa and the Middle East

## Abstract

Snakebite in the Middle East and North Africa (MENA) is a public health problem whose magnitude is not fully known. Several antivenoms are available in these regions, but these formulations are designed for restricted geographical settings. Many countries do not have local production of antivenoms and must access products whose clinical performance has not been demonstrated. We hypothesize that it is possible to unify the treatment for viperid snakebites of MENA in a single antivenom formulation. Hereby we describe the design, development and preclinical evaluation of an antivenom of broad geographical coverage for this region (MENAVip-ICP). We produced this antivenom from the plasma of horses immunized with eight medically important venoms of viperid snake species from MENA. For this, we used a strategy based on two stages: first, immunization of horses with North African (NA) venoms, followed by a second immunization stage, on the same horses, with MENA venoms. We purified antivenoms from both stages: the Anti-NA and the final product Anti-MENA (MENAVip-ICP). Anti-NA was considered as intermediate formulation and was purified with the intention to study the progression of the immunoglobulin immune response of the horses. Antivenoms from both stages neutralized lethal, hemorrhagic, and procoagulant activities of homologous venoms. Compared to Anti-NA, MENAVip-ICP improved the neutralization profile of intravenous lethality and *in vitro* procoagulant activities of venoms. A notable finding was the difference in the neutralization of lethality when MENAVip-ICP was assessed intraperitoneally versus intravenously in the murine model. Intraperitoneally, MENAVip-ICP appears more effective in neutralizing the lethality of all venoms. Furthermore, MENAVip-ICP neutralized the lethal activity of venoms of species from other regions of MENA, Central/East Asia, and Sub-Saharan Africa that were not included in the immunization protocol. Our results showed that MENAVip-ICP neutralizes the main toxic activities induced by viperid MENA venoms at the preclinical level. Consequently, it is a promising product that could be clinically assessed for the treatment of snakebite envenomings in this region.

## Highlights

• An antivenom with broad geographical coverage for the Middle East and North Africa against viperid snake venoms was prepared.
• Horses were initially immunized with venoms of North African snakes, followed by immunization with venoms from Middle East snakes.
• The antivenom, named MENAVip-ICP, neutralizes all evaluated toxic activities of the venoms used in the immunization process.
• MENAVip-ICP neutralizes lethality of heterologous venoms of species of regions beyond the Middle East and North Africa.

## Graphical abstract

**Figure:**
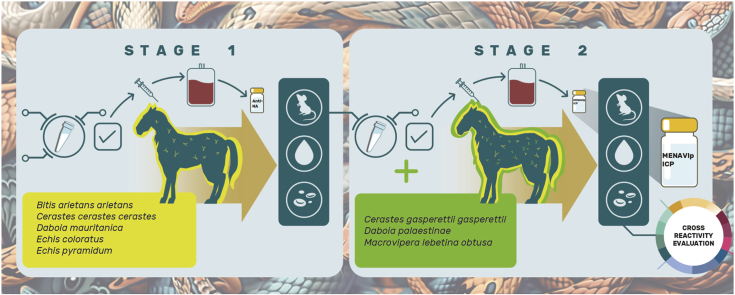

## 1 Introduction

**Fig. 1:**
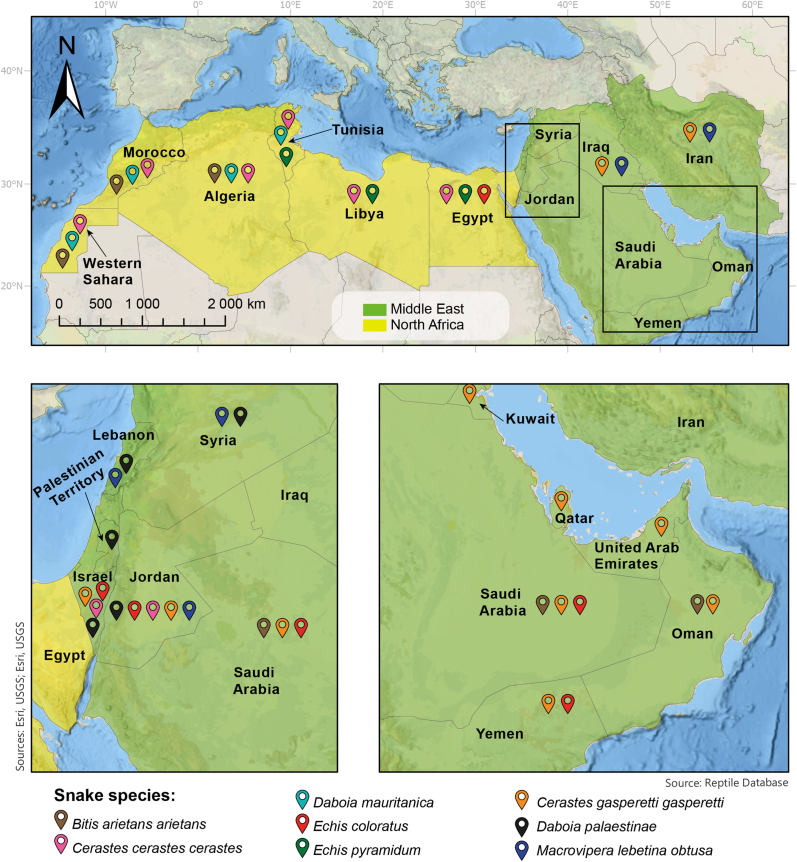
**Geographical distribution of MENA snake species used for the development of MENAVip-ICP.** The upper map presents a general overview of the MENA region. The lower left box shows a zoom of the Levant region in MENA, where Israel, Jordan, Lebanon, the Palestinian Territories, and Syria are located. The lower right box shows a zoom of the Persian Gulf where Kuwait, Qatar and United Arab Emirates are located. Snake distribution was defined according to The Reptile Database.

Countries located in northern Africa and the Middle East are grouped under the acronym **MENA** (**M**iddle **E**ast and **N**orth **A**frica, Fig. 1). About 20 countries integrate this region. The NA region (yellow color in Fig. 1) includes Algeria, Egypt, Libya, Mauritania, Morocco, Tunisia, and West Sahara. Moreover, ME (green color in Fig. 1) includes Bahrain, Israel, Iraq, Islamic Republic of Iran, Jordan, Kuwait, Lebanon, Oman, Palestinian Territories, Qatar, Saudi Arabia, Syria, United Arab Emirates, and Yemen. However, countries in MENA vary according to different sources (Amr et al., 2020); for instance, the World Health Organization (WHO) also includes Cyprus and Turkey in the ME (WHO, 2017). Regardless of the countries included, MENA has a rich and complex diversity of cultures, social dynamics, weather conditions, and biodiversity (Krupp et al., 2009).

Venomous animals are part of the MENA terrestrial biodiversity, and several species of scorpions, spiders, and snakes occur in this region (Jenkins et al., 2021), which can inflict potentially life-threatening envenomings. Accidental encounters between people or domestic animals with snakes of the families Viperidae, Elapidae and Lamprophiidae can result in bites in which the venom enters the body causing local and/or systemic alterations. This event is known as snakebite accident, snakebite envenoming or simply snakebite (Gutiérrez et al., 2017; Malhotra et al., 2021).

In the NA region the accidents are mainly caused by *Bitis arietans* (*Baa*), *Cerastes cerastes* (*Ccc*), *Daboia mauritanica* (*Dm*), *Echis coloratus (Ec*)*, Echis pyramidum* (*Ep*) and *Naja haje (Nh*) (WHO, 2017; Jenkins et al., 2021, Fig. 1). The medically important species in ME are even more diverse: *Baa*, *Ccc*, *Cerastes gasperettii (Cgg*)*, Daboia palaestinae (Dp*)*, Echis borkini*, *Echis carinatus sochureki, Echis coloratus, Echis omanensis*, *Macrovipera lebetina* spp (*Ml*), *Montivipera xanthina* (*Mx*), *Pseudocerastes persicus (Pp*)*, Naja arabica (Na*)*, Naja oxiana* (*No*) and *Atractaspis* spp (WHO, 2017; Amr et al., 2020, Fig. 1).

The precise toll of snakebite envenomation in MENA is largely unknown. Kasturiratne and coworkers (2008) estimated a number of snakebites in all MENA countries that varies between 3000–81 000 per year, with 43–78 deaths, with variations depending on the country. However, recent reports reveal a more drastic scenario (Amr et al., 2020; Malhotra et al., 2021; Jenkins et al., 2021; Abu Baker et al., 2022; Dehghani et al., 2023; Khourcha et al., 2023). Some specific examples of epidemiologic data in MENA region are: Saudi Arabia, 14 700 snakebite cases and 36 deaths in a four-year period (Al-Sadoon et al., 2021); Morocco, 2055 snakebites and 85 deaths from 1999 to 2013 (El Hattimy et al., 2018); Jordan, 121 snakebites clinically attended between 2018 and 2020 (Abu Baker et al., 2022). These reports, and other studies, indicate that snakebite is an underestimated and poorly understood problem in MENA, and that the available clinical therapy is also limited.

The scientifically validated treatment for snakebites is the administration of snake antivenoms obtained from plasma of large animals, mainly horses. For this, equines are hyperimmunized with the snake venoms of interest, and then, their plasma is fractionated by several methods to obtain IgG or IgG fragments-rich formulations, with a high capacity to neutralize venom toxins. These formulations are biopharmaceutical products administered intravenously to patients with clearly established signs and symptoms of envenomation (WHO, 2017).

According to the WHO (https://snbdatainfo.who.int/?data_id=dataSource_1-1835fca1a62-layer-8%3A3413), several formulations of snake antivenoms are available in MENA. Some examples are IPAVIP (Algeria), Gamma-Vip (Tunisia), Vipera palaestinae Antiserum (Israel), and Hexavalent snake venom immunoglobulin and Padra Serum (Islamic Republic of Iran). However, most of these antivenoms are designed for snakebite treatment in specific geographic settings of MENA; moreover, only a few MENA countries possess an installed capacity for antivenom production. Consequently, many countries rely on antivenoms produced with venoms from other geographic areas, which, in many cases, are ineffective against medically important snake venoms in MENA (Abu Baker et al., 2022; Khourcha et al., 2023). Therefore, the design of a broad-spectrum antivenom for MENA, effective in the neutralization of the most relevant snake venoms of this region, would contribute to the improvement of the viperid snakebite envenoming treatment.

In this communication we describe the design and development of MENAVip-ICP, a new snake antivenom with therapeutic potential for the treatment of viperid snakebites in the entire MENA region (Fig. 1). The first step was to carefully select the snake venoms to be included, to ensure extensive geographical coverage. Afterward, we designed the immunization protocol, considering the development of an effective immune response and the horses well-being. Once the antivenom was produced, we determined the preclinical neutralization profile of MENAVip-ICP on the toxic activities of homologous and heterologous snake venoms of medical relevance on these regions.

## 2 Materials and methods

### 2.1 Ethics and animal management

The Institutional Committee for the Care and Use of Laboratory Animals (CICUA) of Universidad de Costa Rica (Act 202–2020) approved all animal procedures used in this study. This study met The International Guiding Principles for Biomedical Research Involving Animals (Bankowski and Howard-Jones, 1985).

Lethal and hemorrhagic activities of snake venoms and their corresponding neutralization tests were evaluated in CD-1 mice (both sexes) weighing between 16 g and 22 g, according to each test (see below). Mice were obtained from the Bioterium of Instituto Clodomiro Picado and handled in Tecniplast Eurostandard Type II 1264C cages, five mice per cage, at 18–24 °C, 60–65% relative humidity, and 12:12 light-dark cycle, with food and water *ad libitum*.

The immunization schedule to produce the antivenom was carried out using three Creole horses. These animals were kept at the experimental farm of the Instituto Clodomiro Picado, with access to water and pasture *ad libitum*. In addition, horses received granulate-enriched food (2 kg/animal/day). Horses were subjected to complete blood counts, blood chemistry analyses, and a general physical examination before the immunization.

According to WHO recommendations (WHO, 2017), during the snake venom immunization schedule, horses were regularly monitored for potential signs of physical and/or behavioral changes. Additionally, complete blood counts were conducted before each booster, and blood chemistry tests were performed at the conclusion of the two stages of immunization (as outlined below) prior to bleeding. Furthermore, any local inflammation or indications of systemic disturbances were meticulously evaluated following each venom boost.

### 2.2 Snake venoms

The following lyophilized venoms were purchased from LATOXAN: *Bitis arietans (Baa), Bitis gabonica (Bg*)*, Bitis nasicornis (Bn*)*, Bitis rhinoceros (Br*)*, Cerastes cerastes (Ccc), Cerastes gasperettii (Cgg), Daboia mauritanica (Dm), Daboia palaestinae (Dp)*, *Echis carinatus sochureki* (*Ecs*)*, Echis coloratus (Ec)*, *Echis leucogaster* (*El*)*, Echis ocellatus* (*Eo*), *Echis pyramidum* (*Ep*), *Macrovipera lebetina obtusa* (*Mlo*) and *Pseudocerastes persicus* (*Pp*). The venoms of *Daboia russelii* (*Drr*), *Echis carinatus* (*Ecc*), *Protobothrops mucrosquamtus* (*Pm*) and *Trimeresurus stejnegeri* (*Ts*) belonged to the venom collection of Instituto Clodomiro Picado and were obtained from India, Sri Lanka and Taiwan, respectively. The geographical origin of the species used in venom collection is depicted in Table 1, Table 4.

**Table 1:** MENA snake venoms utilized in the immunization protocol of MENAVip-ICP.

Geographical Region	Immunization stage	Venom	Abbreviation	Batch a	Geographical Origin of the venom a	Geographical distribution of the species in MENA^b^
NA (North Africa) (yellow color in Fig. 1)	Stage 1: Anti-NA	*Bitis arietans*^c^	*Baa*	322 061	Saudi Arabia, Mali, Kenya, Tanzania	Algeria, Morocco, Western Sahara, Oman, Saudi Arabia
		*Cerastes cerastes*^c^	*Ccc*	201 100	Egypt, Tunisia	Algeria, Egypt, Libya, Morocco, Tunisia, Western Sahara, Israel, Jordan
		*Daboia mauritanica*	*Dm*	112 040	Morocco	Algeria, Morocco, Tunisia, Western Sahara
		*Echis coloratus*^c^	*Ec*	512 191	Egypt	Egypt, Israel, Jordan, Saudi Arabia, Yemen
		*Echis pyramidum*	*Ep*	425 011	Egypt	Egypt, Libya, Tunisia
MENA (Middle East and North Africa) (green color in Fig. 1)	Stage 2: MENAVip-ICP	*Cerastes gasperettii*	*Cgg*	320 102	Saudi Arabia	Iran, Iraq, Israel, Jordan, Kuwait, Oman, Qatar, Saudi Arabia, United Arab Emirates, Yemen
		*Daboia palaestinae*	*Dp*	38 012	Israel	Israel, Jordan, Lebanon, Syria, Palestinian Territories
		*Macrovipera lebetina obtusa*	*Mlo*	420 181	Azerbaijan	Iran, Iraq, Jordan, Lebanon, Syria

a Latoxan information.

b According to: http://reptile-database.reptarium.cz/.

c According to The Reptile Database, these species could be present in both geographic regions. For color codes see Fig. 1.

### 2.3 Venom electrophoretic profile by SDS-PAGE

Electrophoresis was performed according to Laemmli (1970). Venoms, dissolved in non-reducing sample buffer, were loaded on 12% polyacrylamide gels (20 μg/well), and run on a Miniprotean Bio-Rad System (Berkeley, CA, USA). The protein bands were stained with Coomassie blue R-250 in methanol/acetic acid, and then the gel was destained with several changes of destaining solution. Bio Rad molecular weight markers (Berkeley, CA, USA) were used.

### 2.4 Toxic and enzymatic activities of venoms

Lethal and toxic activities of the venoms were determined to define the challenge doses for the neutralization assays.

#### 2.4.1 Determination of median lethal dose (LD_50_)

The median lethal dose was assessed according to WHO (2017). We assessed two administration routes, intravenous (IV) and intraperitoneal (IP). For the IV route, we used mice of 18–22 g and 200 μL of venom solution/mouse; in the case of IP route, we used 16–20 g mice and 500 μL of venom solution/mouse, using 0.12 M NaCl, 0.04 M phosphate, pH 7.2 (PBS) as diluent. Five doses of each venom were evaluated, using five mice per dose. Lethal activity was expressed as LD_50_, which is defined as the dose of venom in which half of the mice die in an observation period of 24h for IV route or 48h for IP route. LD_50_ and the corresponding 95% confidence intervals were calculated by Probits (Finney, 1971) and expressed as μg venom/mouse.

#### 2.4.2 Determination of minimum hemorrhagic dose (MHD)

Hemorrhagic activity was assessed in 18–20 g CD1 mice, according to WHO (2017) recommendations, following the method of Jenkins et al. (2017). We prepared five doses of each venom, dissolved in PBS, and aliquots of 100 μL were injected intradermally, in the abdominal region, to groups of five mice. After 2 h, mice were sacrificed by CO_2_ inhalation, and the magnitude of the hemorrhagic lesions (area and intensity) in the inner side of the skin was measured. The Minimum Hemorrhagic Dose (MHD) was defined as the mass of venom that produced a lesion of 100 hemorrhagic units (HaU).

#### 2.4.3 Determination of minimum procoagulant dose (MPD)

Procoagulant activity on calcified human plasma was determined following the method proposed by O ' Leary and Isbister (2010) and modified by Pla et al. (2020). For each venom, duplicates of ten dilutions, prepared in 25 mM Tris, 137 mM NaCl, 3.4 mM KCl, pH 7.4, were added on 96-well microplates. Afterward, 100 μL of human plasma, containing 4 μL of 0.4 M CaCl_2_ (final concentration 0.015 M), was added to each well. Plates were immediately placed in a microplate reader (Multiskan SkyHigh Microplate Spectrophotometer, Thermo Scientific, Waltham, MA, USA) to record the absorbance at 340 nm. Recordings were carried out every 30 s during a period of 15 min at 37 °C. The Minimum Procoagulant Dose (MPD) was defined as the mass of venom that induces a change in absorbance of 0.1 units within 1 min.

### 2.5 Design and development of MENAVip-ICP

#### 2.5.1 Snake venom selection

For the antivenom design, we selected the most medically important venomous snake species in the Middle East (ME) and North-Africa (NA) based on several sources: 1) the WHO Guidelines on the Production and Control of Snake Antivenom Immunoglobulins (WHO, 2017), 2) the Guidelines on the Management and Prevention of Snakebites in Africa (WHO World Health Organization, 2010), and 3) available clinical reports from various countries in MENA (Abu Baker et al., 2022; Al-Sadoon et al., 2021; Alkaabi et al., 2011; Tirosh-Levy et al., 2019; Essafti et al., 2022; Khourcha et al., 2023; Dehghani et al., 2023). Fig. 1 shows the species selected and their distribution.

#### 2.5.2 Horse immunization schedule and blood extraction

MENAVip-ICP was developed in two stages (Table 1). In the initial stage, we immunized three horses with snake venoms of NA species. These horses received increasing doses of a venom mixture ranging from 0.75 to 4.50 mg across six different boosters. Each booster was administrated every two weeks over a period of nine weeks. Before the injection, the venom mix was emulsified with Montanide ISA 50V2 (SEPPIC, Castres, France, batch 200528019700) following the method described by Arguedas et al. (2022). Subsequently, 1 mL of the emulsified mixture was administered subcutaneously to each horse, alternating injection sites across four points located on the lateral sides of the back, in proximity to lymph nodes, as detailed by León et al. (2011). Following completion of this initial immunization stage, each horse underwent blood extraction, yielding 4 L of blood per animal. The hyperimmune plasma was then separated and stored at 4 °C according to WHO guidelines (2017).

After a six-week rest period, the horses entered the second stage of immunization, during which they were injected with the same snake venoms from North Africa (NA) as well as with venoms from Middle East (ME) species (Table 1). Once again, the horses received increasing emulsified doses of a venom mixture, ranging from 0.50 to 4.00 mg, across six different boosters administered over a nine-week period. At the conclusion of this immunization protocol, a second blood extraction was performed following the previously described method. The entire immunization schedule spanned approximately six months.

#### 2.5.3 Horse plasma fractionation and snake antivenom formulation

We prepared two pools of 5L of plasma (Table 1): Anti-NA and anti-MENA (MENAVip-ICP). These plasma pools were independently precipitated to obtain two IgG formulations, following the caprylic acid fractionation method (Rojas et al., 1994). The following modifications to the original method were added: 1) the plasma pH before precipitation was not adjusted, and 2) a 4% v/v caprylic acid final concentration was used. The final formulations of the two snake antivenoms were adjusted to pH 7.0 and then dialyzed against NaCl 0.9% m/v and phenol 0.2% m/v solution. Finally, both snake antivenoms were concentrated and sterilized by filtration in 0.22 μm pore membranes and bottled in 10 mL glass vials. The physicochemical and microbiological characterization of the antivenoms was conducted in accordance with the Manual of the Quality Control Laboratory, 2023. The parameters assessed included protein concentration, phenol concentration, sodium chloride concentration, endotoxin content, pH, and turbidity.

#### 2.5.4 Neutralization profile of MENAVip-ICP

##### 2.5.4.1 Neutralization of lethal activity

The lethality neutralization test was performed by the IV and IP routes using the same conditions described in section 2.4.1. Different amounts of antivenom were confronted with a constant amount of venom: four LD_50_ for the IP route and five LD_50_ for the IV route (except for *Daboia* spp. venoms, in which case we used three LD_50_ for the IV route due to their high toxicity). For each neutralization test, we assessed five doses of antivenom with five mice per dose. As a positive control a group of three mice received venom incubated with PBS instead of antivenom. The mixtures of venom and antivenom were incubated for 30 min at 37 °C before injection. Neutralization of lethal activity was expressed as median effective dose (ED_50_), which is the mg venom/mL antivenom ratio at which half of mice survived. ED_50_ and the corresponding 95% confidence intervals were calculated by Probits (Finney, 1971).

##### 2.5.4.2 Neutralization of hemorrhagic activity

We prepared mixtures containing a constant amount of venom (5 MHD) and increasing amounts of antivenom. The solutions were incubated for 30 min at 37 °C and then injected into groups of five mice per treatment, as described in section 2.4.2. As positive control, we injected 3 mice with 5 MHD of venom without antivenom. Neutralization was expressed as median effective dose_50_ (ED_50_), defined as the ratio of mg venom/mL antivenom in which the magnitude of the hemorrhagic lesion is reduced to half of the value of the positive control.

##### 2.5.4.3 Neutralization of procoagulant activity

Venoms that exhibited procoagulant activity were confronted with different amounts of snake antivenom, and the mixtures were incubated for 30 min at 37 °C. Then, 100 μL of each solution, containing 2 MPD, were transferred to 96-well microplates, and the procoagulant activity was measured as described in Section 2.4.3. A control of 2 MPD without antivenom was included in each assay. The neutralizing capacity of the snake antivenoms was expressed as the effective dose (ED), expressed as the ratio of mg venom/mL antivenom in which the clotting time is extended three-fold as compared to the venom control.

### 2.6 Statistical analyses

Values of median lethal dose (LD_50_) and its neutralization (ED_50_) were considered different when the 95% confidence intervals (CI) did not overlap. Differences in the Minimal Hemorrhagic Dose (MHD) and Minimal Procoagulant Dose (MPD) were assessed by a Kruskal-Wallis test; values were considered significantly different when p < 0.05. Comparisons between Anti-NA and MENAVip-ICP antivenoms for the neutralization of either hemorrhagic or procoagulant activities were assessed by one-way ANOVA. If required, further analyses were performed, consisting of a general linear model (univariate analysis of variances). Values of p < 0.05 were considered statistically significant.

## 3 Results and discussion

### 3.1 Design and development of MENAVip-ICP

#### 3.1.1 Rationale of venom selection

MENAVip-ICP was designed to neutralize viperid venoms from the MENA regions. Our initial approach was focused on category 1 viperid snake species following the WHO recommendations for the development of a new snake antivenoms (WHO, 2017). Table 1 lists the species of snakes selected and Fig. 1 shows their geographical distribution. For practical reasons, we divided the species in terms of whether they could only be found in either of the regions (NA or ME) (yellow and green colors respectively in Fig. 1). The Reptile Database reports *Baa, Ccc* and *Ec* as present in several countries of NA and ME regions. Nevertheless, these distributions are dissimilar: *Baa* is present in MENA as isolated populations in Morocco, West Sahara, Oman, Saudi Arabia and Yemen (Barlow et al., 2013); meanwhile, Ec is mainly present in ME and only present in North and South Sinai, Egypt in the NA region (Pook et al., 2009); finally, Ccc is distributed through all NA and only in Israel and Jordan in the ME (Al-Sadoon et al., 2021; Essafti et al., 2022).

Venoms from other category 1 viperid species such as *Echis borkini*, *Echis carinatus sochureki, Echis omanensis, Montivipera* spp*,* other subspecies of *Macrovipera lebetina* spp and *Pseudocerastes* spp*,* were not available at the beginning of the immunization protocol, therefore were not included in this first design. However, some of these venoms were tested as heterologous venoms in neutralization lethality tests to assess the paraspecificity of MENAVip-ICP (see data below).

In the design of MENAVip-ICP, we excluded neurotoxic Elapidae and cardiotoxic Lamprophiidae venoms, such as those from species such as *Naja arabica*, *Naja haje*, *Naja oxiana*, and *Atractaspis* spp., which are classified as category 1 venomous snakes in these regions (WHO, 2017). Unlike regions such as Sub-Saharan Africa, Asia, and Oceania, where elapid and viperid species play a prominent role in envenoming epidemiology, the MENA exhibit a lower number of medically significant elapid species compared to viperid species (WHO, 2017; Jenkins et al., 2021). While two lamprophiid species may be medically important in countries of the MENA region (WHO, 2017; Amr et al., 2020), snakebites inflicted by *Atractaspis* spp. are rare (WHO, 2017; Jenkins et al., 2021; Abu Baker et al., 2022).

In addition to the low number of medically important elapid species, it is clinically possible to differentiate between syndromic presentations of viperid and elapid envenomations. Viperid syndrome is usually characterized by local signs and symptoms such as radiating pain, swelling, bleeding from fang puncture wounds, edema, ecchymosis, and myonecrosis. Systemically, individuals bitten by viperids may experience hypotension, severe bleeding, spontaneous bleeding from various anatomic sites, thrombocytopenia, and venom-induced consumption coagulopathy. (Gutiérrez et al., 2017).

In contrast, Elapidic syndrome, as described by MENA *Naja* spp, is characterized by descending flaccid paralysis, beginning with palpebral ptosis and subsequently affecting muscle innervation in the neck, thorax, and limbs. This leads to impairment of swallowing, respiration, and mobility (WHO World Health Organization, 2010; Amr et al., 2020; Abu Baker et al., 2022; Dehghani et al., 2023). This scenario suggests that it could be beneficial for clinical personnel in MENA to have access to separate antivenoms to treat viperid or elapid envenomations.

### 3.2 Horse health status throughout MENAVip-ICP development

During the whole immunization schedule, the three horses were monitored for any signs of pain or anatomic or physiologic alterations after each venom booster. It was observed that the horses showed a good mood, normal physical activity, good appetite, and no sign of aggressiveness during all the experiment time. The only event reported in all horses was mild nervous behavior after the first boost and immediately before the subsequent boosters. This was attributed to a normal behavior in horses not yet habituated to the periodic routine immunizations.

All venom boosters were emulsified with Montanide (Arguedas et al., 2022) to ensure slow delivery of the venom. No signs of systemic envenomation were detected in the three horses. At the local level, we observed the appearance of inflamed areas at the injection sites one day after each booster. However, this inflammation was typically reabsorbed within five to six days. At the end of the schedule, the maximum venom quantity administered to each horse was 4 mg of a mixture of venoms; at this point none of the horses showed signs of envenomation or distress. Moreover, hematological parameters were not altered when compared with their corresponding values before the venom injection (data not shown).

### 3.3 Characterization of MENA snake venoms used to formulate MENAVip-ICP

#### 3.3.1 Electrophoretic profile of snake venoms

**Fig. 2:**
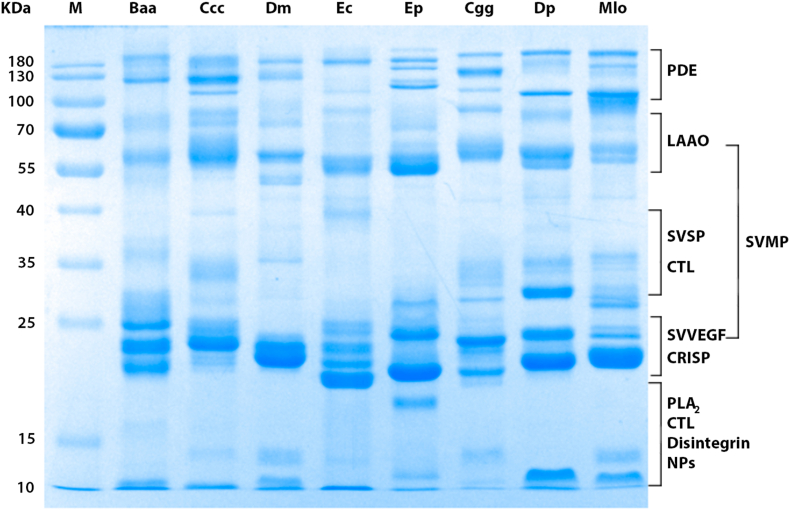
**SDS-PAGE electrophoresis of venoms used in the development of MENAVip-ICP.** Samples were separated in 12% polyacrylamide gel under non reducing conditions (20 μg protein/lane). The families of toxins were labeled according to proteomic studies cited in text.

All the venoms used in the immunization have been previously characterized by proteomic analyses, *Baa* (Juárez et al., 2006; Casewell et al., 2014), *Ccc* (Bazaa et al., 2005; Fahmi et al., 2012; Casewell et al., 2014), *Dm* (Makran et al., 2012), *Ec* and *Ep* (Casewell et al., 2014), *Cgg* (Zaeri et al., 2021), *Dp* (Momic et al., 2011; Senji Laxme et al., 2022) and *Mlo* (Bazaa et al., 2005; Pla et al., 2020). Therefore, we performed an SDS-PAGE to verify that the specific batches of venom used possessed similar protein profiles as previously reported. The electrophoresis patterns of the venoms did not show major variations as compared to those described previously (Fig. 2). Based on the known molecular masses of the various venom protein families, the following components are likely to be present in these venoms: snake venom metalloproteinases (SVMPs), snake venom serine proteinases (SVSPs), phospholipases A_2_ (PLA_2_), C type lectins (CTL), and L-amino acid oxidases (LAO), among others (Damm et al., 2021). The minor variations observed when compared with previous works are expected because snake venoms have interspecies variation in their toxin composition (Chippaux et al., 1991).

#### 3.3.2 Toxicity profile of snake venoms

We characterized the toxicity of the eight venoms used in the immunization schedule by analyzing their lethal, procoagulant and hemorrhagic activities (Table 2), since these are some of the most relevant effects from the clinical point of view (WHO World Health Organization, 2010).

**Table 2:** Lethal, hemorrhagic and procoagulant activities of MENA snake venoms utilized in the immunization scheme.

Geographical Region	Venom	Lethality LD_50_		Hemorrhage MHD	Procoagulant MPD
		I.V. μg	I.P. μg	μg/mouse	μg
North Africa (yellow color in Fig. 1)	*Baa*	11.4 a (10.4–12.5)	21.6 a (14.6–37.4)	0.07 ± 0.01	null up to 100 μg^d^
	*Ccc*	15.1 a (10.9–21.6)	28.6 a (22.1–38.7)	0.10 ± 0.03	0.040 ± 0.000
	*Dm*	6.8 a (4.9–8.9)	17.1 a (13.6–23.2)	0.25 ± 0.10	3.040 ± 1.190 c
	*Ec*	8.3 a (5.0–11.6)	22.1 a (14.8–30.6)	0.31 ± 0.09	0.014 ± 0.000
	*Ep*	21.9 (14.1–33.9)	38.5 (29.8–49.8)	0.16 ± 0.02 b	0.140 ± 0.000
Middle East (green color in Fig. 1)	*Cgg*	16.2 (11.9–23.1)	19.9 (14.6–27.8)	0.17 ± 0.04 b	0.100 ± 0.040
	*Dp*	5.7 a (4.4–7.7)	21.1 a (16.3–28.2)	0.41 ± 0.18	null up to 100 μg^d^
	*Mlo*	18.1 (13.8–25.0)	31.7 (23.5–52.1)	0.17 ± 0.06 b	0.763 ± 0.002 c

LD_50_: dose of venom in which half of the mice die in an observation period of 24h for intravenous (IV) route or 48h for intraperitoneal (IP) route. MHD: the mass of venom that produced a lesion of 100 hemorrhagic units (HaU) 2 h after injection. MPD: the mass of venom that induces a change in absorbance at 340 nm of 0.1 units within 1 min.

a Statistically significant difference between LD_50_ values of the same venom by IV and IP routes.

b All venoms showed statistically significant differences between their MHD values; exceptions are *Ep*, *Cgg* and *Mlo*, which MHD values are similar between them (Kruskal-Wallis: 22.626; df = 7; significance <0.05).

c Venoms with the highest MPD values that are statistically different compared to the other venoms (Kruskal-Wallis: 10.922; df = 5; significance <0.05).

d venoms devoid of procoagulant activity at the highest tested and previously reported as anticoagulant.

Most venoms showed significantly higher lethality by the IV route (Table 2); IP LD_50_/IV LD_50_ ratios were 1.9 for *Baa* and *Ccc*, 2.5 for *Dm*, 2.7 for *Ec* and 3.7 for *Dp*. This tendency to more lethal activity by IV route in MENA viperid venoms was also reported by Oukkache et al. (2014) for *Baa, Ccc* and *Mlo* venoms from Morocco, and could reflect the role of different toxin families according to the route of venom entrance (Kocholaty et al., 1968). However, for the venoms of *Ep*, *Cgg* and *Mlo* the confidence limits of LD_50_ by the two routes overlapped, indicating a similar toxicity.

The most lethal venoms administrated via the IV route include those from *Dm*, *Ec* and *Dp*. In the case of *Dp* venom, its lethality is likely the result of synergistic action of various toxins previously documented (Momic et al., 2011). For instance, the presence of neurotoxic PLA_2_ promotes neurotoxicity, contributing to mice mortality upon IV administration of this venom (Momic et al., 2011). Additionally, this venom exhibits high anticoagulant activity (Op den Brouw et al., 2021, Table 2), which may be linked to the potentiation of its hemorrhagic effects (Youngman et al., 2019). It is worth noting that *Dm* and *Dp* are phylogenetically related (Senji Laxme et al., 2022), therefore both venoms could have a similar mode of action in mice, however, the mechanism of action of *Dm* venom has not been fully characterized (Makran et al., 2012; Chakir et al., 2019). Finally, the high toxicity of *Ec* venom by the IV route could be associated with its high procoagulant potency (Gómez et al., 2022), which may induce intravascular coagulation.

All venoms induced local hemorrhage in the murine model (Table 2). By arranging the venoms from the most to the least hemorrhagic, the following pattern emerged: *Baa* > *Ccc* > *Ep* = *Cgg* = *Mlo* > *Dm* = *Ec* > *Dp* (Kruskal-Wallis: 22.626; df = 7; p < 0.05). This activity is due to the action of SVMPs (Gutiérrez et al., 2017), a family of toxins that is abundant in viperid venoms (Damm et al., 2021, Fig. 2). Moreover, clinically snakebites caused by species of Table 2, are characterized by local and systemic hemorrhage (Amr et al., 2020; Jenkins et al., 2021; Abu Baker et al., 2022; Dehghani et al., 2023).

Procoagulant activity was present in six of the eight venoms (Table 2). The venoms of *Ec, Ccc, Cgg* and *Ep* exert a strong procoagulant effect on human plasma, with an MPD value below 0.15 μg. All these venoms were previously reported to have a high capacity for causing disturbances in the coagulation process (Casewell et al., 2014; Ainsworth et al., 2018 Oukkache et al., 2012; El-Aziz et al., 2020; Jenkins et al., 2021; Zaeri et al., 2021; Gómez et al., 2022). It has been demonstrated the role of SVMPs, SVSPs, PLA_2_ and C-type lectin-like protein in the development of these alterations in viperid venoms (Kini and Koh, 2016; Ainsworth et al., 2018), and, as shown in Fig. 2, these families of toxins are abundant in the venoms used in this study. Venoms of *Mlo* and *Dm* were less procoagulant when compared with the four venoms described above. Both venoms were previously reported as procoagulant due to the action of SVMPs and SVSPs on fibrinogen (Pla et al., 2020; Op den Brouw et al., 2021). *Baa* and *Dp* venoms did not show procoagulant activity on plasma up to 100 μg; both venoms have been described as anticoagulant through mechanisms associated to fibrinogen degradation via SVMPs, an effect that we did not evaluate in this investigation (Youngman et al. (2019), Currier et al., 2010; Kini and Koh, 2016; Nielsen et al., 2016; Youngman et al. (2019); Op den Brouw et al., 2021; Senji Laxme et al., 2022).

Our results show that the venoms used in this study possess important hemorrhagic and, with two exceptions, procoagulant effects, therefore, determining these activities is crucial during the development of a snake antivenom for the MENA region. The preclinical neutralization of such activities can provide guidance on its potential efficacy in the clinical treatment of snakebite victims.

### 3.4 Characterization of antivenoms

The biochemical and microbiological characterization of Anti-NA and MENAVip-ICP is presented in Table 3. All parameters meet the antivenom specifications established by the Quality Control Laboratory of the Instituto Clodomiro Picado for industrially produced antivenoms. The recorded content of endotoxin does not have a specified range parameter, as it is determined based on the dosage required for clinical management of snakebites; in this instance, the antivenoms are still in the development stage. However, based on clinical treatment protocols for viperid snakebite cases in Costa Rica, the endotoxin data obtained (Table 3) would initially allow the administration of 10 vials of antivenom to a person.

**Table 3:** Physicochemical and microbiological characterization of Anti-NA and MENAVip-ICP antivenoms.

Parameter	Stage 1:Anti-NA	Stage 2: MENAVip-ICP	Specification^a^
Total Protein (g/dL)	6.80 ± 0.05	6.60 ± 0.10	≤12
Sodium chloride (g/dL)	0.85 ± 0.01	0.80 ± 0.02	0.75–1.00
Phenol (g/dL)	0.21 ± 0.05	0.22 ± 0.03	0.20–0.25
pH	7.00 ± 0.06	6.95 ± 0.03	6.50–7.50
Turbidity (NTU)	25 ± 2	26 ± 1	≤50
Endotoxin (EU/mL)	≤3	≤3	Not defined b

g/dL: grams per deciliter of solution. NTU: nephelometric turbidity units. EU/mL: endotoxin units per milliliter of solution.

a According to the Manual of Quality Control Laboratory, Instituto Clodomiro Picado, Universidad de Costa Rica.

b Endotoxin content has no specification because it is defined according to clinical dose (see text).

Anti-NA was considered as intermediate formulation and was purified with the intention to describe the progression of the immune response of the horses, and their immune reactivity when confronted against homologous and heterologous (*Cgg*, *Dp* and *Mlo*) venoms.

### 3.5 Preclinical efficacy of MENAVip-ICP

#### 3.5.1 Lethal activity neutralization

**Fig. 3:**
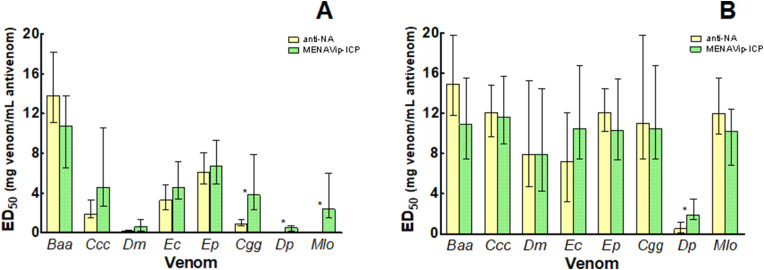
**Neutralization of lethal activity of homologous venoms by Anti-NA and MENAVip-ICP. A.** Results of neutralization when using the IV route. **B.** Results of neutralization when using the IP route. Neutralization is expressed as ED_50_ in mg of venom/mL of antivenom. Color codes: yellow  for Anti-NA and green  for MENAVip-ICP. Venom abbreviations are in Table 1. * Significant enhancement of ED_50_ values of MENAVip-ICP compared to Anti-NA.

Fig. 3 shows the lethality neutralizing profile of the MENAVip-ICP in its two stages: Anti-NA and Anti-MENA, both by the IV and IP routes (3A and 3B respectively).

##### 3.5.1.1 Stage 1: Anti-NA

Anti-NA neutralized lethality of all homologous venoms, *i.e., Baa, Ccc, Dm, Ec* and *Ep* venoms at the end of stage 1, when tested by the IV route. However, ED_50_ varied according to each venom (Fig. 3A). The IV ED_50_ against *Dm* venom was the lowest value with 0.23 mg of venom/mL of Anti-NA; meanwhile neutralization of *Baa* reaches a value of 13.8 mg of venom/mL of Anti-NA. The values of IV ED_50_ for *Ccc*, *Ec* and *Ep*, were 1.90, 3.30 and 6.10 mg venom/mL antivenom, respectively (Fig. 3A–Supplementary Table 1).

We also assessed the efficacy of Anti-NA to neutralize the lethality of ME venoms, with the aim of following the immune response against all the venoms during the immunization protocol. Anti-NA neutralized the venom of *Cgg,* evidencing the presence of cross-reactive immunoglobulins in the antivenom, probably anti-*Ccc* immunoglobulins generated during this first stage of immunization. In contrast, venoms of *Dp* and *Mlo* were not neutralized by Anti-NA when tested by the IV route (Fig. 3A).

Different results were observed when evaluating the neutralization of lethality by the IP route. Interestingly, Anti-NA showed a good neutralization against all assessed venoms, either homologous or heterologous (Fig. 3B). It is striking the magnitude of the increase in ED_50_ values by the IP route as compared to the IV route: 6-fold for *Ccc*, 2-fold for *Ec* and *Ep*, 12-fold for *Cgg;* the highest change was seen *with Dm* venom, with 35-fold increased. *Baa* venom is the only one that did not show an ED_50_ value increase with the change in the administration route (Fig. 3A and B); however, independently of the administration route, *Baa* ED_50_ reflects a high capacity of Anti-NA to neutralize this venom.

##### 3.5.1.2 Stage 2: MENAVip-ICP

When stage 2 of the immunization protocol was completed, MENAVip-ICP evidenced a significant enhancement in its ED_50_ values, assessed by the IV route, for the venoms of *Cgg*, *Dp* and *Mlo* (Fig. 3A–Supplementary Table 1). For the other 5 venoms, no statistically significant changes in the ED_50_ values between the first and second stage of immunization were observed, however, there were a tendency to increase for *Ccc, Ec* and *Dm* venoms, and a tendency to decrease for *Baa* venom (Fig. 3A). When the neutralization of lethality is assessed by the IP route (Fig. 3B), the ED_50_ value of MENAVip-ICP was not modified for any of the venoms in comparison to Anti-NA, the only exception being an increase of the neutralizing efficacy on *Dp* venom. MENAVip-ICP, similarly to Anti-NA, evidenced a higher neutralizing potency by the IP route for all venoms evaluated; the only exception is *Baa* venom, that has the same ED_50_ for both routes.

The change in the ED_50_ lethality values depending on the route of venom administration was previously reported for other viperid venoms in IP/IV neutralization (Kocholaty et al., 1968; Solano et al., 2010); our results show a higher efficacy when using the IP route than the IV route (Fig. 3). The reason behind the differences of ED_50_ values by IV vs IP route obtained by us and others, is not clearly understood. Kocholaty et al. (1968) and Solano et al. (2010) proposed that this phenomenon probably reflects the roles in lethality of different set of toxins depending on the route, and consequently the neutralization by each route, depends on different neutralizing antibodies.

In the realm of antivenom production, a thorough understanding of the wide array of toxins capable of inducing lethality is imperative for both quality control personnel and manufacturers. Additionally, they must consider the potential variation in toxin neutralization outcomes based on the evaluation route utilized in the murine model. Moreover, this assessment provides invaluable insights into the immunoglobulin response triggered by toxins acquired through horse immunization, Consequently, it aids in making informed decisions regarding antivenom production for clinical application. Such knowledge guides the establishment of specifications to determine the preclinical effectiveness of an antivenom against specific venoms.

The selection of a route for routinely assessing venom lethality and its neutralization in a murine model for quality control in antivenom production is ultimately determined by each center. The WHO guidelines for antivenoms accept both routes of injection for evaluating antivenom efficacy (WHO, 2017). However, the criteria for choosing a specific route are not clearly defined. Moreover, relying solely on murine lethality neutralization for assessing antivenom preclinical efficacy has been criticized (Kocholaty et al., 1968; Chippaux et al., 1991; Barlow et al., 2009; Solano et al., 2010; Richards et al., 2012; Oukkache et al., 2014). Therefore, evaluating other clinically relevant effects allows for a more comprehensive assessment of preclinical efficacy (WHO, 2017). To better understand the neutralization capacity of MENAVip-ICP, we conducted additional assays to evaluate the neutralization of hemorrhagic and procoagulant activities.

#### 3.5.2 Neutralization of hemorrhagic activity

**Fig. 4:**
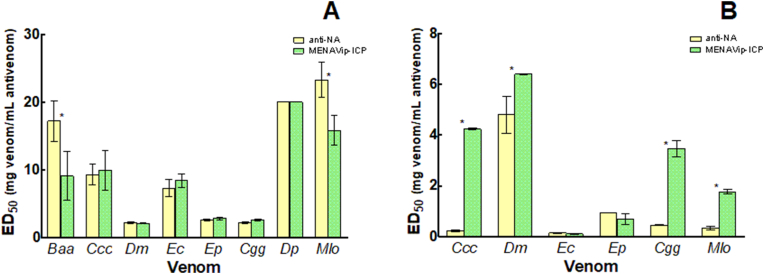
**Neutralization of hemorrhagic and procoagulant activities of homologous venoms by Anti-NA and MENAVip-ICP. A.** Neutralization of hemorrhagic activity expressed as ED_50_ in mg of venom/mL of antivenom. * Significant difference between ED50 values of Anti-NA and MENAVip-ICP (F = 81.683; df = 15; p < 0.001). **B.** Neutralization of procoagulant activity expressed as ED in mg of venom/mL of antivenom. * Significant difference between ED values of Anti-NA and MENAVip-ICP (F = 165–072; df = 11; p < 0.001). Color codes: yellow  for Anti-NA and green  for MENAVip-ICP. Venom abbreviations are in Table 1.

The results of neutralization of hemorrhagic activity for all venoms by Anti-NA or MENAVip-ICP are shown in Fig. 4A and Supplementary Table 2. Both antivenoms were highly effective in the neutralization of this effect in all venoms tested.

Anti-NA neutralized the hemorrhagic activity of the five venoms used in the first stage of immunization; in addition, Anti-NA was able to neutralize hemorrhage induced by the three venoms of ME (Fig. 4A). Noteworthy, this neutralization reaches ED_50_ values of approximately 20 mg of venom/mL of Anti-NA for *Baa*, *Dp* and *Mlo* venoms. For the venoms of *Ccc* and *Ec* the values of ED_50_ are close to 10 mg of venom/mL Anti-NA, a value that also evidences a high efficacy. The other venoms were also neutralized, however the ED_50_ values are around 2 mg of venom/mL Anti-NA. Interestingly, after the second stage of immunization (MENAVip-ICP) the ED_50_ values did not change (F = 0.773; d f = 59; p = 0.383) for most of the venoms (Fig. 4A); furthermore, there seems to be a decrease in neutralization hemorrhagic activity for *Baa* and *Mlo* venoms (F = 81.683; df = 15; p < 0.001), however, the values of ED_50_ are still above 9 mg venom/mL of MENAVip-ICP.

The high ED_50_ values evidence that both antivenoms possess a high titer of neutralizing antibodies against the hemorrhagic SVMPs present in the venoms of NA and ME. Moreover, our results suggest that MENAVip-ICP has polyvalency against hemorrhagic SVMPs of MENA venoms, and therefore could contribute to the neutralization in the clinical setting of other pathological effects associated to the action of these toxins, such as dermonecrosis, myonecrosis, coagulopathies and edema (Gutiérrez et al., 2016). Antivenomics studies have demonstrated that antivenoms generally have high efficacy in the recognition of SVMPs (Gutiérrez et al., 2014).

#### 3.5.3 Neutralization of procoagulant activity

The efficacy of Anti-NA and MENAVip-ICP antivenoms to neutralize the procoagulant activity of the venoms is depicted in Fig. 4B and Supplementary Table 3.

Anti-NA antivenom achieved neutralization of the homologous venoms *Ccc*, *Dm*, *Ec* and *Ep*. Moreover, it also neutralized the ME venoms *Cgg* and *Mlo* (Fig. 4B). This neutralization, expressed as ED_50_ in mg of venom/mL of Anti-NA, showed different neutralization capacities according to the venom: *Ccc* 0.22; *Dm* 4.74; *Ec* 0.14; *Ep* 0.94; *Cgg* 0.44 and *Mlo* 0.33 mg of venom/mL of Anti-NA (Supplementary Table 3). Even though Anti-NA neutralizes the coagulant activity of these venoms, the neutralizing efficacy is low as compared to neutralization of lethal and hemorrhagic effects, which suggests that immunoglobulins against procoagulant toxins are either in low titer or have a low neutralizing capacity. Exceptions are the venoms of *Dm* and *Ep*, which are neutralized with ED values above 0.9 mg/mL with Anti-Na antivenom. We did not assess venoms of *Baa* and *Dp* because they are devoid of procoagulant activity in our *in vitro* test (Table 2).

At the end of the second stage of immunization, when we had the first version of MENAVip-ICP, the neutralization of the procoagulant activity improved for most venoms (Fig. 4B). Compared with anti-NA, MENAVip-ICP shows a noteworthy increase in its efficacy on four of six venoms: 19-fold for *Ccc*, 1.4-fold for *Dm*, 8-fold for *Cgg* and 5-fold for *Mlo* (F = 165.072; df = 11; p < 0.001). This enhancement of the neutralizing capacity of MENAVip-ICP against procoagulant toxins of NA venoms is probably due to the incorporation of ME venoms in the immunization mixture. Furthermore, it is possible that the increase in immunoglobulin titers against procoagulant toxins is associated with the improvement of the lethality neutralization observed for the venoms of Cgg*, Dp* and *Mlo* by the IV route (Fig. 1A).

The only venoms in which the neutralization capacity did not increase were *Ec* and *Ep* (F = 3.860; df = 1; p = 0.062). The result for *Ec* venom agrees with previous report that show the difficulty of rabbit serum Anti-*Ec* to neutralize the procoagulant activity of this venom. (Gómez et al., 2022). It is interesting that MENAVip-ICP achieves a high neutralization capacity of *Ec* venom lethality and hemorrhage, but not for its procoagulant activity. Therefore, a deeper understanding of the procoagulant toxins of this venom is needed to increase the titer of neutralizing immunoglobulins.

Finally, it is worth mentioning that in the previously described neutralization of toxic activities, such as lethality, hemorrhagic, and procoagulant effects, horses received twice as many NA venom boosters as ME venom boosters (see Section 2.5.2). Whether this variation influenced the results obtained needs to be explored in future studies.

### 3.6 Neutralization of heterologous venoms by MENAVip-ICP

The ability of MENAVip-ICP to neutralize lethality of heterologous snake venoms was also evaluated. For this, we selected venoms from species inhabiting countries in ME, Sub-Saharan Africa, and Central/East Asia. The LD_50s_ of these venoms by the IP and IV routes were determined (Supplementary Table 4).

Table 4 summarizes the heterologous venoms studied, their country of origin and the ED_50_ values obtained when venoms were incubated with the MENAVip-ICP. Venoms from *E. c. sochureki and E. leucogaster* from the MENA regions were fully neutralized by the IP and IV routes in mice, meanwhile *P. persicus* venom was only neutralized by the IP route. Venoms of Asian snakes, geographically distant from ME, specifically from India/Sri Lanka (*D. russelii* and *E. c. carinatus*) and Taiwan (*P. mucrosquamatus, T. stejnegeri*) were also neutralized by IP and IV routes. A striking result was the capacity of MENAVip-ICP to neutralize by IP and IV routes the venoms of *B. gabonica, B. rhinoceros* and *E. ocellatus* from Sub-Saharan Africa; and the venom of *B. nasicornis* by the IP route.

**Table 4:** Lethality cross-neutralization of MENAVip-ICP on several heterologous snake venoms.

Geographical Region	Venom a (Batch)	Geographical origin of the venom	Geographical distribution of the species b	I.V. ED_50_ mg/mL c	I.P. ED_50_ mg/mL d
**MENA**	*Echis carinatus sochureki* (800.081)	Pakistan a	Iran, Iraq, United Arab Emirates, southeast Arabian Peninsula	2.1 (1.4–3.7)	10.8 (7.2–20.1)
	*Echis leucogaster* (Batch 425.011)	Mali a	Algeria, Morocco	6.5 (4.6–8.9)	8.0 (6.1–11.1)
	*Pseudocerastes persicus* (504.100)	Pakistan a	Iran, Iraq, Kuwait, Saudi Arabia, Oman, United Arab Emirates, Jordan, Syria	< 1.6	9.2 (6.3–12.7)
**Asia countries outside ME**	*Daboia russelii* (MR-245)	India/Sri Lanka e	Pakistan, India, Nepal, Sri Lanka, Bangladesh, Bhutan	0.8 (0.5–1.2)	0.4 (0.2–0.8)
	*Echis carinatus* (MR-246)	India/Sri Lanka e	India, Sri Lanka	3.4 (2.4–5.1)	7.6 (3.6–11.6)
	*Protobothrops mucrosquamatus*	Taiwan e	Bangladesh, China, China, Laos, Myanmar, Taiwan, Thailand, Vietnam	5.4 (3.9–11.0)	3.0 (2.0–4.5)
	*Trimeresurus stejnegeri* (MR-125)	Taiwan e	China, India, Lao People's Democratic Republic, Myanmar, Taiwan, Vietnam)	2.2 (1.3–3.6)	4.7 (2.8–6.4)
**Sub-Saharan Africa**	*Bitis gabonica* (722.090)	Burundi and Tanzania a	Benin, Nigeria, Chad, Republic of South Sudan, Central African Republic, Cameroon, Uganda, Equatorial Guinea, Congo, Democratic Republic of the Congo, Angola, Zambia, Kenya, Rwanda, Gabon, Tanzania, Zimbabwe, Mozambique, Republic of South Africa, Malawi, Togo	1.1 (0.7–2.1)	6.6 (5.2–8.3)
	*Bitis nasicornis* (801.001)	Burundi and West Africa a	Republic of South Sudan, Kenya, Uganda, Angola, Democratic Republic of the Congo, Congo, Gabon, Cameroon, Guinea, Nigeria, Benin, Togo, Ghana, Ivory Coast, Liberia, Sierra Leone, Guinea, Central African Republic, Tanzania, Zambia	< 1.6	6.4 (4.8–9.8)
	*Bitis rhinoceros* (701.070)	Ghana a	Guinea, Guinea-Bissau, Liberia, Sierra Leone, Ivory Coast, Ghana, Togo	4.2 (2.8–11.5)	9.5 (7.8–12.5)
	*Echis ocellatus* (200.171)	Cameroon, Mali and Ghana a	Burkina Faso, Mali, Mauritania, Ghana, Ivory Coast, Togo, Benin, Nigeria, Senegal, Guinea, Gambia, Niger	7.2 (4.5–15.3)	9.4 (6.9–12.7)

ED_50_: ratio of mg venom/mL of antivenom in which half of the mice survive in an observation period of 24h for intravenous (IV) route or 48h for intraperitoneal (IP) route.

a Latoxan catalogue information.

b According to: http://reptile-database.reptarium.cz/.

c Challenge dose 5 LD_50_.

d Challenge dose 4 LD_50_.

e Venom belonged to the venom collection of Instituto Clodomiro Picado and were obtained from India, Sri Lanka and Taiwan.

These observations suggest that our immunization protocol, using a mixture of venoms from species distributed along MENA (Fig. 1 and Table 1), generated a wide immunoglobulin repertoire against relevant toxins involved in the lethality induced by the assessed heterologous venoms in the mouse model. It is likely that MENAVip-ICP is capable of neutralizing other toxic effects in addition to the lethality produced by these venoms, such as hemorrhage, hemostasis disorders and local necrosis, an issue that awaits future studies. Our findings suggest that MENAVip-ICP could be considered as an option for the treatment of envenomings produced by species of Table 4. Moreover, it is necessary to expand the analysis of the preclinical efficacy of this new antivenom against venoms of other venomous snakes present in MENA region, such as *Daboia deserti, Vipera latastei, Echis omanensis, Echis borkini, Echis khosatzkii, Pseudocerastes fieldi* and *Eristocophis macmahonii,* all of which are medically relevant (WHO, 2017). Based on the results of this investigation, three additional batches of MENAVip-ICP have been manufactured. Following quality control assessment, these batches are currently undergoing stability studies.

## 4 Concluding remarks

The development of new antivenoms should consider the need to generate products with wide neutralization scope, able to be used in the clinical setting to treat envenomings by the medically most relevant species in wide geographical regions. Consequently, the design, development, and assessment of preclinical efficacy of a new antivenom, should be based on information on topics as: snakes species present in a defined geographic area, epidemiological snakebite data, historical clinical management of envenomings in this area, projections of efficacy on homologous and heterologous venoms of high medical impact and the preclinical evaluation of the neutralization of specific toxic/enzymatic effects of venom targets. Moreover, since horses are the actual bioreactors for immunoglobulin antivenom production, they should be strictly monitored throughout the immunization schedule and bleeding process to ensure their health status.

The design and development of MENAVip-ICP described in this study meets most of these requirements. The resultant antivenom developed for viperid snakebite treatment in MENA neutralized the lethal, hemorrhagic and procoagulant activities of all assessed homologous venoms. Although Anti-NA is capable of neutralizing most of the venoms evaluated, the inclusion of ME venoms improved the efficacy of the antivenom against specific venoms. Furthermore, MENAVip-ICP exerts a good neutralization of the lethal activity of heterologous venoms from MENA and of other Sub-Saharan Africa and Central/East Asian venoms. It is likely that the immunizing venom mixture used contained a variety of toxins that elicited a wide immunoglobulin repertoire that targets many different epitopes of the toxins present in these venoms.

Finally, since there is geographical variation in venom composition within snake species and some of the species included in this study have a wide geographical distribution, it is important to assess MENAVip-ICP against a broader spectrum of snake venoms from several geographical locations of MENA not included in this work to further evaluate the spectrum of preclinical efficacy of this antivenom. Our observations indicate that MENAVip-ICP is a novel and potentially valuable therapeutic alternative for the management of snakebite envenoming in these regions.

## Ethical statement

The authors declared that all procedures involving animals were approved by the Institutional Committee for the Care and Use of Laboratory Animals (CICUA) of Universidad de Costa Rica (Act 202–2020).

## CRediT authorship contribution statement

**Álvaro Segura:** Writing – review & editing, Writing – original draft, Project administration, Methodology, Investigation, Conceptualization. **Edwin Moscoso:** Writing – review & editing, Methodology, Investigation. **Deibid Umaña:** Writing – review & editing, Methodology, Investigation. **Mariángela Vargas:** Writing – review & editing, Methodology, Investigation, Conceptualization. **Andrés Sánchez:** Writing – review & editing, Methodology, Investigation, Conceptualization. **Andrés Hernández:** Writing – review & editing, Methodology, Investigation, Conceptualization. **Gina Durán:** Methodology, Investigation. **Mauren Villalta:** Writing – review & editing, Methodology, Investigation, Conceptualization. **Aarón Gómez:** Writing – review & editing, Methodology, Formal analysis. **María Herrera:** Writing – review & editing, Methodology, Investigation, Conceptualization. **Mauricio Arguedas:** Methodology. **José María Gutiérrez:** Writing – review & editing, Funding acquisition. **Guillermo León:** Writing – review & editing, Funding acquisition.

## Declaration of competing interest

The authors declare the following financial interests/personal relationships which may be considered as potential competing interests:All authors work in the Instituto Clodomiro Picado, Universidad de Costa Rica, where the horses were maintained and immunized, the antivenoms purified and all preclinical experiments carried out. If there are other authors, they declare that they have no known competing financial interests or personal relationships that could have appeared to influence the work reported in this paper.

## Data Availability

Data will be made available on request.
