# Supplementary material for: Design, development and preclinical assessment of MENAVip-ICP, a new snake antivenom with potential coverage of species in the Middle East and North Africa regions
Source: Toxicon X. 2024 Aug 30;24:100206. doi: 10.1016/j.toxcx.2024.100206 (PMC11403368; doi:10.1016/j.toxcx.2024.100206)
Supplement: Multimedia component 1 [file mmc1.docx]

**Supplementary table 1. Neutralization of lethal activity of homologous venoms by Anti-NA and MENAVip-ICP.**

| **Injection Route** | **Venom** | **Batch ^1^** | NA  (North Africa)  (yellow color in Fig. 1) | MENA  (Middle East and North Africa)  (green color in Fig. 1) |
| --- | --- | --- | --- | --- |
| **I.V. ED_50_**  **mg venom/mL antivenom**  **(Challenge dose: 5 LD_50_)** | *Bitis arietans arietans* | 322 061 | 13.80  (11.10-18.20) | 10.47  (6.46-33.6) |
|  | *Cerastes cerastes cerastes* | 201 100 | 1.90  (1.50-3.0) | 4.60  (2.70-10.0) |
|  | *Daboia mauritanica* **^2^** | 112 040 | 0.23  (0.15-0.3) | 0.60  (0.18-1.7) |
|  | *Echis coloratus* | 512 191 | 3.30  (2.30-4.0) | 4.60  (3.40-7.0) |
|  | *Echis pyramidum* | 425 011 | 6.10  (4.90-8.0) | 6.68  (4.87-9.8) |
|  | *Cerastes gasperettii gasperettii* | 320 102 | 0.90  (0.70-1.0) | 3.81 **^3^**  (2.26-7.0) |
|  | *Daboia palaestinae* **^2^** | 38 012 | ˂ 0.17 | 0.45 **^3^**  (0.18-0.4) |
|  | *Macrovipera lebetina obtusa* | 420 181 | ˂ 0.84 | 2.43 **^3^**  (1.50-5.7) |
| **I.P. ED_50_**  **mg venom/mL antivenom**  **(Challenge dose: 4 LD_50_)** | *Bitis arietans arietans* | 322 061 | 14.90  (11.80-23.0) | 10.90  (7.50-15.0) |
|  | *Cerastes cerastes cerastes* | 201 100 | 12.10  (9.70-14.0) | 11.60  (9.00-15.0) |
|  | *Daboia mauritanica* | 112 040 | 8.00  (4.70-27,.0) | 7.90  (4.30-14.0) |
|  | *Echis coloratus* | 512 191 | 7.20  (3.20-12.0) | 10.50  (7.50-16.0) |
|  | *Echis pyramidum* | 425 011 | 12.10  (10.20-14.0) | 10.30  (7.40-15.0) |
|  | *Cerastes gasperettii gasperettii* | 320 102 | 11.00  (7.50-19.0) | 10.50  (7.50-16.0) |
|  | *Daboia palaestinae* | 38 012 | 0.50  (0.10-1.0) | 1.90  (1.10-3.0) |
|  | *Macrovipera lebetina obtusa* | 420 181 | 12.00  (9.90-15.0) | 10.20  (6.80-21.0) |

ED_50_: ratio of mg venom /mL antivenom in which half of the mice survived in an observation period of 24h for intravenous (IV) route or 48h for intraperitoneal (IP) route. LD_50_: dose of venom in which half of the mice survived in an observation period of 24h for intravenous (IV) route or 48h for intraperitoneal (IP) route. **^1^** Latoxan catalogue number. **^2^** vs 3LD_50_. **^3^** ED_50_ values are significantly different for the same venom assessed with Anti-NA or MENAVip-ICP.
