# Supplementary material for: Design, development and preclinical assessment of MENAVip-ICP, a new snake antivenom with potential coverage of species in the Middle East and North Africa regions
Source: Toxicon X. 2024 Aug 30;24:100206. doi: 10.1016/j.toxcx.2024.100206 (PMC11403368; doi:10.1016/j.toxcx.2024.100206)
Supplement: Multimedia component 2 [file mmc2.docx]

**Supplementary Table 2. Neutralization of hemorrhagic activity of homologous venoms by Anti-NA and** **MENAVip-ICP.**

|  | **Immunization stage** | **Stage 1:**  **Coverage only for North Africa snake venoms**  **(Anti-NA)** | **Stage 2:**  **Expansion of coverage to snake venoms from the Middle East**  **(MENAVip-ICP)** |
| --- | --- | --- | --- |
| **Geographical Region** | **Venom** | **ED_50_ mg/mL** | **ED_50_ mg/mL** |
| NA (North Africa)  (yellow color in Fig. 1) | *Baa* | 17.2 ± 3 | 9.1 ± 3.6 |
|  | *Ccc* | 9.3 ± 1.5 | 9.9 ± 2.9 |
|  | *Dm* | 2.2 ± 0.1 | 2.1 ± 0.1 |
|  | *Ec* | 7.3 ± 1.3 | 8.4 ± 1.0 |
|  | *Ep* | 2.6 ± 0.1 | 2.8 ± 0.2 |
| MENA (Middle East and North Africa)  (green color in Fig. 1) | *Cgg* | 2.2 ± 0.1 | 2.6 ± 0.1 |
|  | *Dp* | 20 ± 0 | 20 ± 0 |
|  | *Mlo* | 23.3 ± 2.3 | 15.8 ± 2.2 |

ED_50_: Median effective dose expressed as ratio of venom mg/mL of antivenom in which the magnitude of the hemorrhagic lesion is reduced to half of the value of the positive control challenged with 5 Minimum Hemorrhagic Doses (MHD) of venom (see Materials and Methods for details).
