# Supplementary material for: Design, development and preclinical assessment of MENAVip-ICP, a new snake antivenom with potential coverage of species in the Middle East and North Africa regions
Source: Toxicon X. 2024 Aug 30;24:100206. doi: 10.1016/j.toxcx.2024.100206 (PMC11403368; doi:10.1016/j.toxcx.2024.100206)
Supplement: Multimedia component 3 [file mmc3.docx]

**Supplementary Table 3. Neutralization of procoagulant activity of homologous venoms by Anti-NA and** **MENAVip-ICP.**

|  | **Immunization stage** | **Stage 1:**  **Coverage only for North Africa snake venoms**  **(Anti-NA)** | **Stage 2:**  **Expansion of coverage to snake venoms from the Middle East**  **(MENAVip-ICP)** |
| --- | --- | --- | --- |
| **Geographical Region** | **Venom** | **ED mg/mL** | **ED mg/mL** |
| NA  (North Africa)  (yellow color in Fig. 1) | *Baa* | Not determined **^1^** | |
|  | *Ccc* | 0.22 ± 0.03 | 4.24 ± 0.05 |
|  | *Dm* | 4.74 ± 0.73 | 6.41 ± 0.00 |
|  | *Ec* | 0.14 ± 0.01 | 0.10 ± 0.01 |
|  | *Ep* | 0.94 ± 0.00 | 0.67 ± 0.21 |
| MENA  (Middle East and North Africa)  (green color in Fig. 1) | *Cgg* | 0.45 ± 0.02 | 3.44 ± 0.31 |
|  | *Dp* | Not determined **^1^** | |
|  | *Mlo* | 0.34 ± 0.07 | 1.77 ± 0.09 |

ED: neutralizing effective dose expressed as the ratio of mg venom/mL antivenom, in which the clotting time is extended three-fold as compared to the venom control challenged with 2 Minimal Procoagulant Dose. MPD: defined as the mass of venom that induces a change in absorbance of 0.1 units within 1 min.

**^1^** Neutralization was not determined because snake venom did not show procoagulant activity up to 100 µg.
