# Supplementary material for: Design, development and preclinical assessment of MENAVip-ICP, a new snake antivenom with potential coverage of species in the Middle East and North Africa regions
Source: Toxicon X. 2024 Aug 30;24:100206. doi: 10.1016/j.toxcx.2024.100206 (PMC11403368; doi:10.1016/j.toxcx.2024.100206)
Supplement: Multimedia component 4 [file mmc4.docx]

**Supplementary Table 4. IV and IP lethal activities of heterologous venoms from MENA, Asia countries (Outside ME), and Sub-Saharan Africa.**

| **Geographical Region** | **Venom** | **Batch** | **I.V. LD_50_**  **µg venom/200 µL/mouse** | **I.P. LD_50_**  **µg venom/500 µL/mouse** |
| --- | --- | --- | --- | --- |
| **MENA**  **(Middle East and North Africa)** | *Echis carinatus sochureki* | 800.081 **^1^** | 43.7  (32.8-104.6) | 47.7  (39.0-58.1) |
|  | *Echis leucogaster* | 425.011 **^1^** | 30.3  (21.9-41.1) | 39.2  (26.4-52.2) |
|  | *Pseudocerastes persicus* | 504.100 **^1^** | 24.5  (17.2-48.4) | 52.3  (44.9-65.9) |
| **Asia countries outside ME**  **(Middle East)** | *Daboia russelii russelii* | MR-245 **^2^** | 6.3  (4.4-9.1) | 21.8  (10.7-39.7) |
|  | *Echis carinatus carinatus* | MR-246 **^2^** | 9.5  (7.1-13.6) | 16  (9.7-21.5) |
|  | *Protobothrops mucrosquamatus* | MT-981216 **^2^** | 21.8  (18.5-25.7) | 40.5  (31.2-62.1) |
|  | *Trimeresurus stejnegeri* | MR-125 **^2^** | 8.2  (5.6-16.2) | 18.4  (11.7-28.9) |
| **Sub-Saharan Africa** | *Bitis gabonica* | 722.090 **^1^** | 20.6  (18.1-23.5) | 28.2  (18.7-35.9) |
|  | *Bitis nasicornis* | 801.001 **^1^** | 19.8  (17.2-22.9) | 33.1  (19.3-47.4) |
|  | *Bitis rhinoceros* | 701.070 **^1^** | 17.9  (15.1-20.4) | 27.3  (23.5-32.0) |
|  | *Echis ocellatus* | 200.171 **^1^** | 18.3  (17.0-19.7) | 31.2  (21.1-49.1) |

LD_50_: dose of venom in which half of the mice survive in an observation period of 24h for intravenous (IV) route or 48h for intraperitoneal (IP) route. **^1^** Latoxan catalogue number. **^2^** Instituto Clodomiro Picado internal code
